# Cutaneous/subcutaneous *RREB1::MRTFB* fusion-positive extra-glossal mesenchymal neoplasm—two cases expanding the anatomical spectrum of an emerging entity

**DOI:** 10.1007/s00428-025-04383-0

**Published:** 2025-12-26

**Authors:** Miroslav Důra, Martina Baněčková, Tomáš Rozkoš, Michal Michal, Michael Michal, Liubov Kastnerová

**Affiliations:** 1 Bioptical Laboratory, Ltd., Pilsen, Czech Republic; 2 Department of Pathology, Charles University Faculty of Medicine in Pilsen, Pilsen, Czech Republic; 3 The Fingerland Department of Pathology, Charles University Faculty of Medicine in Hradec Kralove and University Hospital Hradec Kralove, Hradec Kralove, Czech Republic; 4 Cytopathos, Ltd., Bratislava, Slovakia

**Keywords:** *RREB1::MRTFB* fusion, Extra-glossal mesenchymal neoplasm, Ectomesenchymal chondromyxoid tumor

## Abstract

*RREB1::MRTFB* fusion-positive extra-glossal mesenchymal neoplasm is a recently recognized tumor so far mostly described in the head and neck area and in the mediastinum. At least some of these neoplasms are potentially related to ectomesenchymal chondromyxoid tumor of the tongue since they share an identical gene fusion and overlapping morphological features in some cases. Herein we describe for the first time two cases with *RREB1::MRTFB* fusion located in the skin and subcutis. The cases occurred in 36-year-old male with a cutaneous mass on the nose and in 65-year-old woman with a large subcutaneous mass involving the lower leg. Histopathologically, both cases consisted of bland ovoid cells in a myxoid stroma. Immunohistochemically, one of the two cases showed diffuse S100 positivity. *RREB1::MRTFB* fusion was confirmed in both cases. In summary, the two reported cases expand the anatomical spectrum and improve our understanding of this rare emerging entity.

## Introduction

Ectomesenchymal chondromyxoid tumor (EMCMT) is an extremely rare tumor occurring almost exclusively on the tongue. The vast majority of tested cases were shown to harbor *RREB1::MRTFB* (formerly called *MKL2*) gene fusion [5].

*RREB1::MRTFB* fusion-positive extra-glossal mesenchymal neoplasms represent a potentially even rarer subset, predominantly reported in the head and neck region or in the superior mediastinum [8]. To date, involvement of cutaneous or subcutaneous sites has not been documented. While some reported cases have exhibited overlapping morphological features with EMCMT, due to a broader morphological spectrum, the exact relationship of these tumors to EMCMT is currently uncertain. Herein, we report two cases arising in cutaneous/subcutaneous locations, thereby expanding the known anatomical distribution of *RREB1::MRTFB* fusion-positive extra-glossal mesenchymal neoplasm.

## Case presentations

**Fig. 1:**
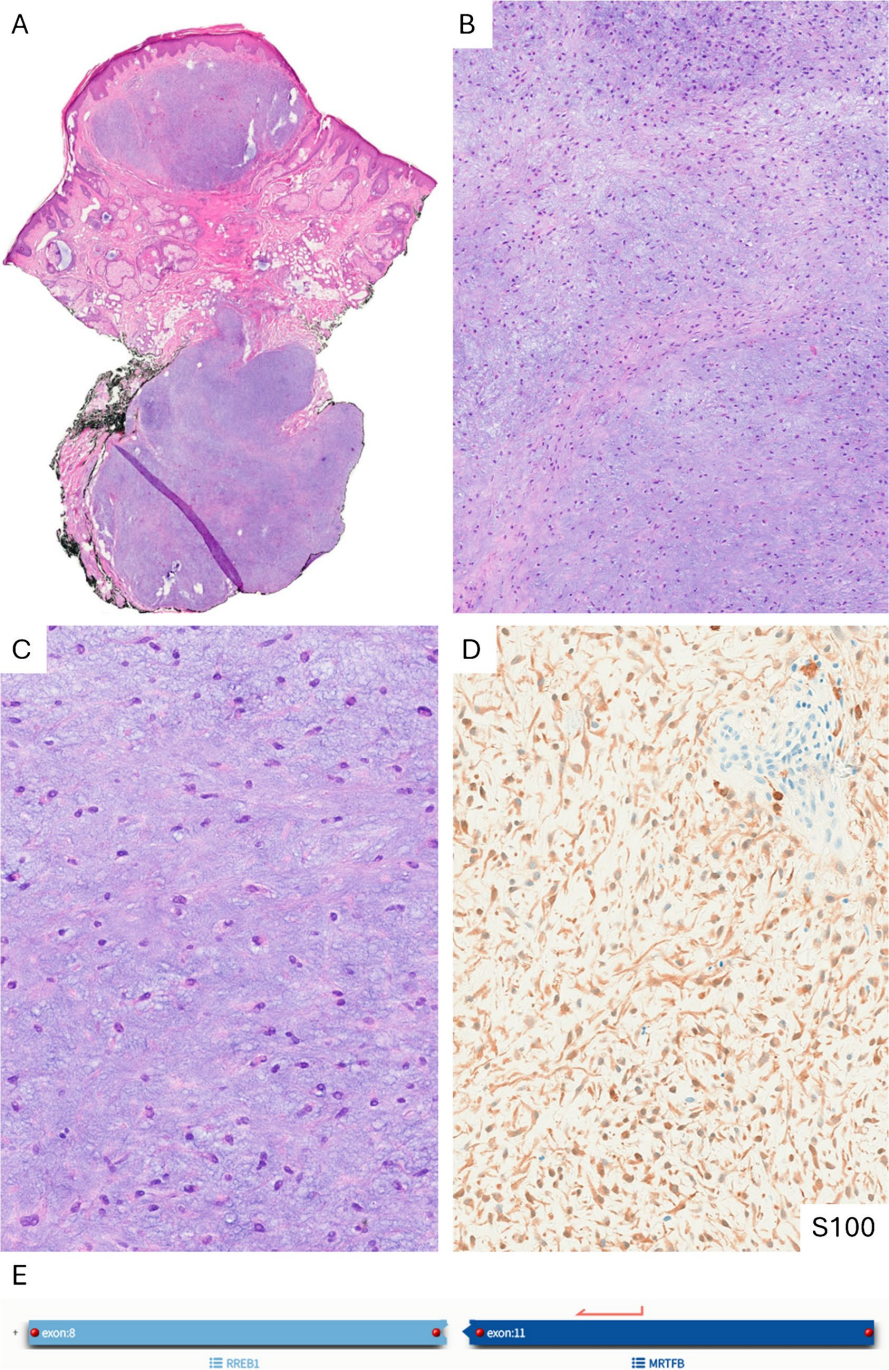
Case 1. The tumor was a multilobulated non-encapsulated mass located in the dermis but extending into the subcutis (**A**). It was composed of bland ovoid cells set in bluish staining myxoid stroma. Neither mitoses nor necrosis were observed (**B**, **C**). The tumor cells were diffusely positive for S100 protein (**D**). Archer FusionPlex RNA sequencing kit detected an in-frame *RREB1* (exon 8)::*MRTFB* (exon 11) gene fusion (**E**)

The first case was a routine biopsy specimen. The patient was a 36-year-old male with a 5 mm cutaneous mass on the dorsal aspect of the nose which was incompletely excised. Histopathologically, the tumor was a multilobulated non-encapsulated mass located in the dermis but extending into the subcutis (Fig. 1A). It was composed of bland ovoid cells set in bluish staining myxoid stroma (Fig. 1B-C). Neither mitoses nor necrosis were observed. The tumor cells were immunohistochemically diffusely positive for S100 protein (Fig. 1D) whereas other markers (ERG, SOX10, SOX9, desmin, EMA, HMB45, cytokeratins AE1/3, CD34 and smooth muscle actin) were negative. An in-house customized version of Archer FusionPlex RNA sequencing kit performed as previously described [18] detected an in-frame *RREB1* (exon 8)*::MRTFB* (exon 11) gene fusion (Fig. 1E). The reference transcripts were *RREB1* NM_001003698.4 and *MRTFB* NM_014048.4. Genomic breakpoints (hg19) were located at chr6:7211942 and chr16:14339385. Complete excision was recommended. The patient was lost for follow-up.

**Fig. 2:**
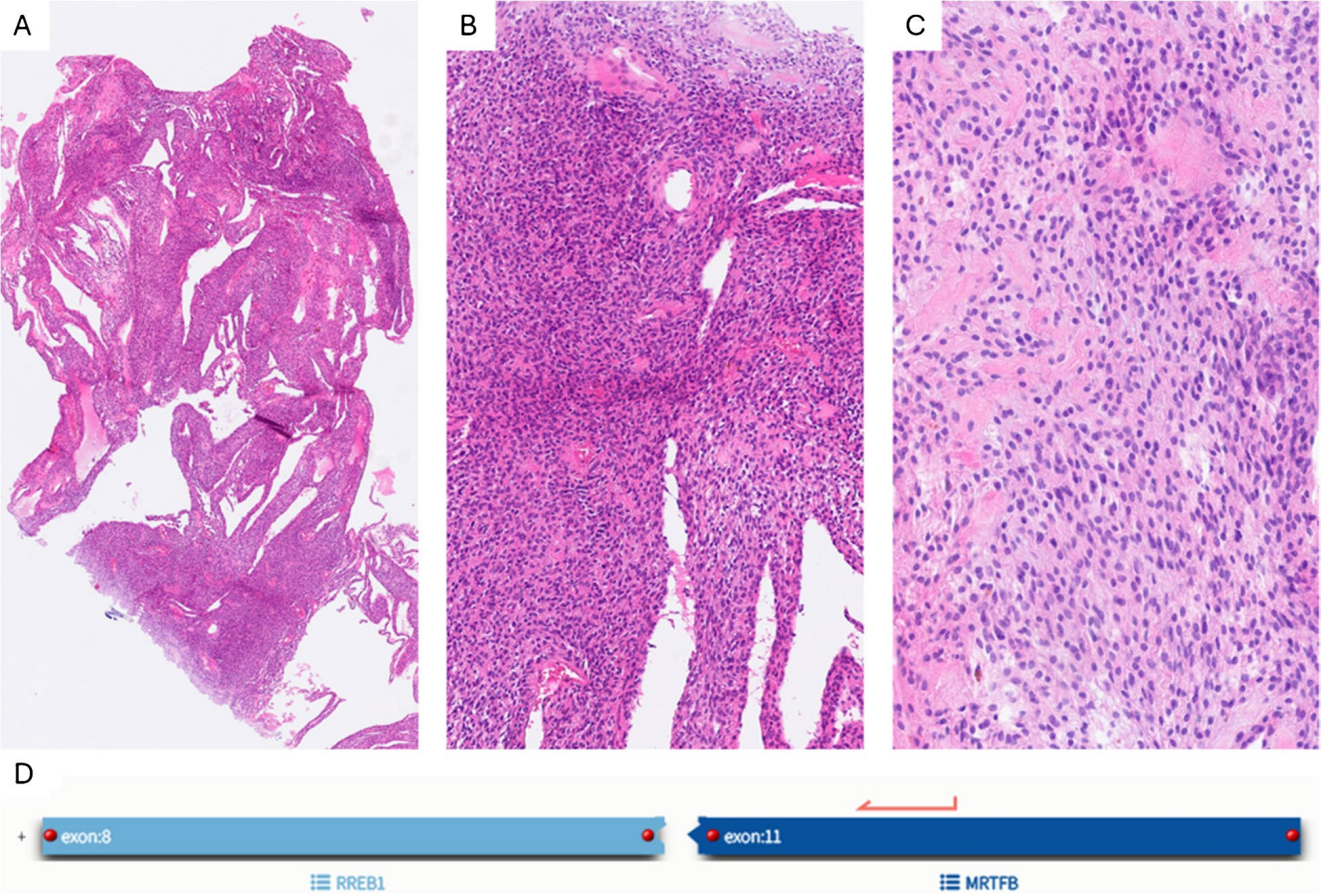
Case 2. The tumor was partially cystic (**A**) and consisted of solid sheets of bland monomorphic ovoid cells without mitotic activity and without necrosis which were set in a myxohyalinized stroma. Occasional hyalinized vessels were observed (**B**, **C**). Archer FusionPlex RNA sequencing kit detected an in-frame *RREB1* (exon 8)::*MRTFB* (exon 11) gene fusion (**D**)

The second case was received in consultation. The patient was a 65-year-old woman with a large subcutaneous mass involving the lower leg, infiltrating neurovascular bundles and causing walking impairment. The diagnostic excision measuring 25 mm in diameter was submitted in fragments. Morphologically, the tumor was partially cystic (Fig. 2A) and consisted of solid sheets of bland monomorphic ovoid cells without mitotic activity and without necrosis which were set in a myxohyalinized stroma. Occasional hyalinized vessels were observed (Fig. 2B-C).

The tumor revealed weak, patchy nuclear and partially cytoplasmic positivity for STAT6 and weak nuclear positivity of MDM2. Other markers (S100, DDIT3, HMGA2, EMA, HMB45, CD34, SMA, AE1/AE3, ERG, MyoD1, PU.1, CD163, desmin, SSX, SS18-SSX) were negative. Based on the vague morphological similarity and immunophenotype, *GLI1*-rearranged mesenchymal tumor and solitary fibrous tumor were considered in the differential diagnosis. Using the same RNA sequencing platform, we also detected an in-frame *RREB1* (exon 8)*::MRTFB* (exon 11) gene fusion (Fig. 2D). Genomic breakpoints (hg19) were identical as in case 1, located at chr6:7211942 and chr16:14339385.

The patient refused subsequent treatment and was lost for follow-up.

## Discussion

EMCMT was described in 1995 by Smith et al., who published 19 cases of this new entity and coined the term EMCMT [19]. Clinically, the tumor is slowly growing, firm, usually painless mass ranging from several millimeters to 5 cm, located almost exclusively on the anterior dorsal aspect of the lingual submucosa. There is no gender predilection, and the median age is approximately 40 years. Nevertheless, EMCMT could occur at any age including in pediatric patients [21].

The histogenesis of the EMCMT is not well established. Originally, it was postulated the tumor originates from the neural crest because of the almost exclusive lingual location (hence the name “ectomesenchymal” related to the ectomesenchyme of first branchial arch origin). This theory is supported by the expression of markers of undifferentiated embryonic stem cells detected by RT-PCR analysis (OCT 3/4, SOX2, Nanog, MAP2 and CD105 mRNAs) [22]. Nevertheless, since other cases similar to EMCMT have been described in several extra-glossal locations, the theory is questionable [10]. The relationship to myoepithelial neoplasms was also proposed [12]. Currently, similarly to other fusion-associated mesenchymal tumors with no clear line of differentiation, WHO classifies EMCMT as a tumor of uncertain histogenesis. The vast majority of EMCMT harbor *RREB1::MRTFB* gene fusion [5, 6]. Although rare cases revealed *EWSR1* rearrangement by FISH, this finding is of unclear significance since RNA-sequencing was not performed and the presence of a gene fusion was thus not confirmed [2].

Tumors with the same *RREB1::MRTFB* gene fusion were also reported at extra-glossal locations, most of them occurring in the head and neck area (hard palate, jaw, retro- or parapharyngeal space), while only a few cases occurred elsewhere in the body (Table 1) [1, 4]. The descriptive term *RREB1::MRTFB* fusion-positive extra-glossal mesenchymal neoplasm was coined for these tumors by Agaimy et al. [1].

**Table 1:** Clinicopathological features of *RREB1::MRTFB* fusion-positive extra-glossal mesenchymal neoplasm described in the literature

	Described by	Age	Sex	Diameter (mm)	Location	Morphology	IHC +	IHC-
1	Siegfried et al. [17]	53	M	35	Retro-/parapharyngeal	Monomorphic spindle cells with scant stroma	SMA, S100, desmin, myogenin	h-caldesmon, SOX10, EMA, p63, MUC4, AE1/AE3, CD34, STAT6, MDM2
2	Makise et al. [8]	25	F	56	Neck/superior mediastinum left	Ovoid cells in fibrous to hyaline stroma	S100, GFAP, SMA, EMA, AE1/AE3, ER	Desmin, myogenin, SOX10, HMB45, MUC4, STAT6, CD34, BCOR, NKX2-2
3	Makise et al. [8]	73	F	43	Superior mediastinum left	Storiform proliferation of bland spindle cells in fibrous stroma	S100, GFAP, SMA, EMA, AE1/AE3, pan-TRK, ER	AE1/AE3, desmin, myogenin, CD34, STAT6, MUC4, SOX10, GLUT1, claudin-1, MDM2, DOG1, SSTR2A, ALK
4	Bubola et al. [3]	37	F	30	Body of mandible right	Spindle cells with storiform pattern in myxohyaline stroma	SMA, S100, CD56, desmin, GFAP	SOX10, AE1/AE3, chromogranin, calponin
5	Mechtersheimer et al. [10]	73	F	35	Posterior middle nasal turbinate right	Monomorphic spindle cells in collagenous stroma	SMA, S100, EMA, CD34	Desmin, myogenin, GFAP, STAT6, AE1/AE3
6	Agaimy et al. [1]	61	M	80	Inguinal/proximal thigh left	Perineurioma-like whorls of spindle cells in fibromyxoid stroma	CD34, EMA (focal), claudin-1 (focal)	Pankeratin, STAT6, SMA, desmin, GLUT1, S100, SOX10, GFAP, ERG, MUC4, INI1 retained
7	Agaimy et al. [1]	36	F	200	Presacral region	Lobular proliferation of ovoid cells in chondromyxoid stroma	S100, CD68, EMA (focal)	Pankeratin, Brachyury, claudin-1, GFAP, MUC4, CD34, SOX10, INI1 retained
8	Agaimy et al. [1]	28	F	NA	Jaw (follicular cyst?)	Lobules of primitive chondromyxoid tissue	S100, CD56 (focal), SATB2 (weak)	Pankeratin, ERG, ALK, Brachyury, MUC4, pan-TRK, SSTR2A, desmin, MDM2
9	Agaimy et al. [1]	28	M	60	Parapharyngeal space	Cellular proliferation of monomorphic spindle/round cells	Synaptophysin, GFAP, CD56, CD99 (focal), PAX8	MUC4, NUT, pan-TRK, STAT6, SS18, ALK, EMA, p63, CK19, TLE1, S100, SMA, SOX10, CD34, CK34BetaE12, desmin, myogenin, MyoD1, WT1, chromogranin-A, calcitonin, TTF1
10	Agaimy et al. [1]	18	M	33	Posterior nasopharyngeal wall	Nested proliferation of monomorphic ovoid and focally spindled cells	MyoD1, desmin (focal), myogenin (focal)	GFAP, S100, beta-catenin, CD34, CD117, CD3, CD19, CD45
11	Midey et al. [11]	57	F	90	Intracardiac	Cellular spindle proliferation in myxoid stroma	S100, synaptophysin, INSM1	Epithelial, muscular, vascular, and melanocytic markers (unspecified)
12	Deng et al. [4]	20	F	44	Left plantar region	Spindle to ovoid cells in myxoid stroma	S100, GFAP, CD56, SMA (weak focal), TFE3 (weak)	Pan-cytokeratin, CD34, ERG, HMB45, Melan-A, p63, SOX10, NSE
13	Current Case	36	M	5	Skin of the nose	Bland ovoid cells in myxoid stroma	S100	ERG, SOX10, SOX9, desmin, EMA, HMB45, AE1/AE3, CD34, SMA
14	Current Case	65	F	"large infiltrative tumor"	Lower leg	Bland ovoid cells in myxohyalinized stroma	STAT6 (weak), MDM2 (weak)	S100, DDIT3, HMGA2, EMA, HMB45, CD34, SMA, AE1/AE3, ERG, MyoD1, PU.1, CD163, desmin, SSX, SS18-SSX

EMCMT is morphologically usually well circumscribed, consisting of a mixture of ovoid and spindled cells in chondromyxoid stroma. Invasion into the adjacent skeletal muscle could be found. In contrast, while some cases of *RREB1::MRTFB* fusion-positive extra-glossal mesenchymal neoplasm show very similar morphological features as EMCMT, the morphological spectrum seems to be broader. Agaimy et al. described 5 cases including two cases located in the soft tissue of the inguinal and presacral region [1]. Microscopically, all cases revealed fibromyxoid stroma with variable cytoarchitecture, including perineurioma-like whorls and storiform pattern in one case and the presence of large polygonal granular cells in the other. Mitotic activity was low in all cases. Our above-described cases revealed analogous histomorphology, i.e., bland ovoid cells in myxoid or myxohyalinized stroma.

The immunohistochemical results were heterogenous; the majority of cases expressed GFAP and S100. The tumors cells were variably positive for cytokeratins, p63, SMA, EMA, desmin, CD34, CD56, CD57, and SOX10 [1].

Overall, the relationship between EMCMT and *RREB1::MRTFB* fusion-positive extra-glossal mesenchymal neoplasm is unclear. However, given the overlapping morphology of some cases, it seems plausible that at least a subset of the latter represents an extraglossal counterpart of EMCMT. Subsequent studies ideally utilizing methylomic and/or transcriptomic data are needed to clarify the relationship between these tumors.

Given the broad morphological spectrum, the differential diagnosis of *RREB1::MRTFB* fusion-positive extra-glossal mesenchymal neoplasm occurring in the skin is extensive. Nevertheless, the main entities necessary to exclude are apocrine mixed tumor (AMT), myoepithelial neoplasms of soft tissue, *GLI1*-altered mesenchymal tumors and perineurioma. AMT could be differentiated by the presence of the ductal structures and the presence of *PLAG1* or *HMGA2* fusions which can be detected either by surrogate immunohistochemical markers or by molecular genetics [9]. Myoepithelial neoplasms of soft tissue typically express some of the so-called myoepithelial markers (S100, SOX10, EMA, and cytokeratins) and show *EWSR1* or *FUS* fusions with variable partners [20]. *GLI1*-altered mesenchymal tumors morphologically present as multinodular masses composed of monomorphic epithelioid or spindle cells often with a nested glomoid architecture and a rich capillary network. The tumor shows a variable expression of S100 and SMA. Genetically, the tumor is defined by either *GLI1* gene fusion or amplification and GLI1 immunohistochemistry can be used as a surrogate marker [7, 13–16]. In addition, the *GLI1*-amplified subset frequently overexpresses MDM2 and/or STAT6 [7, 16]. Perineuriomas typically show a fascicular or whorling proliferation of bland spindle cells combined with immunohistochemical positivity of perineurial markers such as EMA, claudin-1, GLUT1 and CD34 which tend to highlight long cytoplasmic processes of the tumor cells.

Due to a limited number of published cases, the biological potential of *RREB1::MRTFB* fusion-positive extra-glossal mesenchymal neoplasm is not yet fully established. However, none of the 7 reported cases with follow-up information (length 3–27 months, median 10 months) has metastasized. Therefore, a simple surgical excision is currently considered a curative treatment, although rare local recurrences have been described primarily in incompletely resected cases [3].

In summary, we reported two cases of *RREB1::MRTFB* fusion-positive extra-glossal mesenchymal neoplasm at a novel cutaneous and subcutaneous location, thus expanding the anatomical spectrum of this rare emerging entity.
